# First-Episode Medication-Naive Major Depressive Disorder Is Associated with Altered Resting Brain Function in the Affective Network

**DOI:** 10.1371/journal.pone.0085241

**Published:** 2014-01-09

**Authors:** Xiaocui Zhang, Xueling Zhu, Xiang Wang, Xiongzhao Zhu, Mingtian Zhong, Jinyao Yi, Hengyi Rao, Shuqiao Yao

**Affiliations:** 1 The Medical Psychological Institute of the Second Xiangya Hospital, Central South University, Changsha, Hunan, China; 2 Center for Functional Neuroimaging, Department of Neurology, University of Pennsylvania, Philadelphia, Pennsylvania, United States of America; 3 School of Psychology, South China Normal University, Guangzhou, Guangdong, China; 4 Department of Psychology, Sun Yat-Sen University, Guangzhou, Guangdong, China; Yale University School of Medicine, United States of America

## Abstract

**Background:**

Major depressive disorder (MDD) has been associated with abnormal structure and function of the brain's affective network, including the amygdala and orbitofrontal cortex (OFC). However, it is unclear if alterations of resting-state function in this affective network are present at the initial onset of MDD.

**Aims:**

To examine resting-state function of the brain's affective network in first-episode, medication-naive patients with MDD compared to healthy controls (HCs).

**Methods:**

Resting-state functional magnetic resonance imaging (rs-fMRI) was performed on 32 first-episode, medication-naive young adult patients with MDD and 35 matched HCs. The amplitude of low-frequency fluctuations (ALFF) of the blood oxygen level-dependent (BOLD) signal and amygdala-seeded functional connectivity (FC) were investigated.

**Results:**

Compared to HC, MDD patients showed reduced ALFF in the bilateral OFC and increased ALFF in the bilateral temporal lobe extending to the insular and left fusiform cortices. Enhanced anti-correlation of activity between the left amygdala seed and the left OFC was found in MDD patients but not in HCs.

**Conclusions:**

Reduced ALFF in the OFC suggests hypo-functioning of emotion regulation in the affective network. Enhanced anti-correlation of activity between the amygdala and OFC may reflect dysfunction of the amygdala-OFC network and additionally represent a pathological process of MDD.

## Introduction

Major depressive disorder (MDD) is a common psychiatric disorder, typically characterized by pervasive despondency and failure to suppress negative thought, as well as specific cognitive and behavioral alterations [1]. In China, MDD is highly prevalent and constitutes a pressing public health problem, with yearly increases in morbidity and a high risk of mortality [2]. The underlying pathophysiology of this disorder remains unclear. However, a growing body of evidence indicates involvement of prefrontal cortex (PFC) and amygdala dysfunction in the pathophysiology of MDD.

The orbitofrontal cortex (OFC), a sub-region of the PFC, plays an essential role in executive control of information processing and behavioral expression by inhibiting neural activity associated with contextually irrelevant, unwanted, or uncomfortable (e.g. painful) information, sensations, and actions [3]. The OFC and the amygdala, being important parts of the affective network, are involved in the emotional processing of mental states [4]–[6]. Previous neuroimaging studies have revealed abnormal structure and function of the OFC and amygdala in patients with MDD. For instance, evidence from positron emission tomography (PET) showed decreased metabolism in the left OFC in MDD patients [7]–[9], and other neuroimaging studies have shown reduced gray matter volume in the OFC in MDD patients [10]–[12]. In addition, activity in the OFC correlates inversely with the severity of depression in patients with MDD [8], [13]. Conversely, increased amygdala activation has been observed in patients with MDD [14]–[16]. It has been posited that the OFC has a top-down inhibitory effect on the amygdala [17]–[19], and the amygdala-OFC network has been associated with regulation of negative affect, including anger [20]–[22]. Hyperactivity in the amygdala has been inversely associated with OFC reactivity during the suppression of negative emotions [23], and abnormal amygdala-OFC functional connectivity has been observed in depressed patients [24], [25]. There is a convergence of evidence pointing to dysfunction of the OFC, amygdala, or their interaction in the pathophysiology of depression.

Resting-state functional magnetic resonance imaging (rs-fMRI) has been attracting increased attention since Biswal and colleagues first reported the presence of spatially coherent activities in the blood oxygen level-dependent (BOLD) signal [26]. Using rs-fMRI, researchers can assess regional and neural circuitry function at rest in the absence of external tasks; moreover, this approach can be implemented relatively easily in clinical studies [27], [28]. Two parameters calculated from the BOLD signal have been widely used in rs-fMRI study, namely the amplitude of low-frequency (<0.08 Hz) fluctuations (ALFF) and functional connectivity (FC). The ALFF during resting state are considered to be physiologically meaningful and reflective of spontaneous neural activity [29]–[31]. These spontaneous low-frequency fluctuations bear numerous similarities to fluctuations of neural metabolic, hemodynamic, and neurophysiological parameters [32], [33]. Functional connectivity has been defined as “the temporal correlation of a neurophysiological index measured in different brain areas” [34], and can measure the signal synchronicity of low-frequency fluctuation activity among different brain areas [26], which can provide information about the intrinsic brain network organization as well as network dysfunction. ALFF and FC characterize different aspects of resting-state brain functions. ALFF reflect regional amplitude of spontaneous fluctuations in specific regions, while FC measures functional integration among different brain areas. These complementary parameters can help to better characterize brain pathophysiology of MDD patients. Previous studies have used resting scans to investigate ALFF or FC in MDD patients; however, to our knowledge, there is no single fMRI study integrating ALFF and FC to investigate MDD patients' resting brain function. Several recent studies have used rs-fMRI to investigate changes in resting brain function in MDD patients but reported inconsistent findings [35]–[37]. For example, Jiao et al. found increased frontal cortex activity, including right dorsolateral prefrontal cortex, bilateral triangular inferior frontal gyrus and orbital inferior gyrus in adolescents with MDD [35] compared with healthy controls. In constrast, Wang et al. observed decreased frontal cortex activity, including in the left dorsolateral prefrontal cortex and bilateral medial orbitofrontal cortex in first-episode, treatment-naive patients with MDD [36]. Aberrant resting-state FC has also been observed in patients with MDD. For example, using a region of interest (ROI) analysis, Tang et al. detected decreased FC between the amygdala and left ventral PFC in treatment-naive patients with MDD [38]. In a multivariate pattern analysis study, Zeng et al. demonstrated that MDD patients showed altered activity in several resting-state networks, including the affective network, relative to healthy controls [39]. Using independent component analysis, other researchers have found decreased connectivity of the amygdala and left anterior insula [40] and abnormal FC in the default mode network [41] in treatment-naive patients with MDD. These inconsistent findings may be due to the different methodologies and rs-fMRI parameters used, as well as different characteristics of participants, including age, gender, illness severity and treatment history.

In the present study, we applied rs-fMRI to a relatively large sample of first-episode, medication-naive young adult patients using both ALFF and FC analyses in order to better elucidate the alterations of resting brain function in MDD patients. We tested the following two hypotheses: compared with healthy controls, MDD patients will show (1) reduced ALFF in the OFC and amygdala, as well as (2) abnormal FC between the amygdala and the OFC.

## Methods

### Participants

A total of 32 first-episode, medication-naive young adult patients with MDD (age: 18–24 years, mean 20.53 years; 18 females) were recruited from the psychiatric clinic at Second Xiangya Hospital of the Central South University in Changsha, China. A group of 35 healthy controls (HCs) matched for sex, age, and education (age: 18–24 years, mean 20.97 years; 17 females) were recruited from two local two universities (Table 1). Two expert psychiatrists confirmed the diagnoses based on the DSM-IV criteria for MDD [42]. All patients were experiencing their first episode of depression and had never received antipsychotic medications or other medications before at the time of the MRI recording. Both groups met the following criteria: (1) right-handed undergraduate students; (2) no current or previous psychiatric disorder (for the MDD group, no other major psychiatric illnesses, including bipolar disorder); (3) no family history of psychotic disorders or personality disorders; (4) no previous head trauma with loss of consciousness; (5) no persistent headaches; (6) no history of alcohol or substance abuse; (7) no current or previous use of electroconvulsive therapy or psychotropic medications; (8) no neurological illnesses, including stroke and dementia.

**Table 1:** Demographic information of study subjects.

Characteristic	MDD patients (n = 32)	Healthy controls (n = 35)	*p* value
Gender: male/female	14/18	18/17	.656
Age, years: mean (s.d.)	20.53 (1.78)	20.97 (1.29)	.255
Education, year: mean (s.d.)	13.88 (0.87)	13.97 (0.86)	.650
CES-D score: mean (s.d.)	38.03 (6.68)	16.20 (6.69)	<.001

MDD  =  major depressive disorder; CES-D  =  Center for Epidemiological Studies Depression Scale; s.d.  =  standard deviation.

Depressive symptoms in participants of both groups were assessed by applying the Center for Epidemiological Studies Depression Scale (CES-D) [43], an instrument that has been confirmed to have a high degree of reliability and validity in China [44].

This study was approved by the Ethics Committee of the Second Xiangya Hospital of Central South University, China, and all participants gave written informed consent to participate.

### Functional MRI resting state paradigm

The participants were directed to remain motionless and relax with their eyes closed without falling asleep, and not to think of anything particular during the MRI scanning. After the scanning session, participants were asked whether they had fallen asleep during the scan. Patients who responded positively or ambiguously were excluded from the study.

### Data acquisition

Magnetic resonance images were acquired using a 1.5 T Siemens Magnetom Symphony scanner at the Magnetic Resonance Center of the Third Xiangya Hospital of Central South University in Changsha, China. Participants wore a standard head coil fitted with foam padding to minimize head movement and diminish scanner noise. Resting-state fMRI images were acquired axially with an echo-planar imaging (EPI) sequence with the following parameters: TR/TE = 2000/40 ms, 26 slices, 64×64 matrix, 90° flip angle, 240-mm FOV, and 5-mm section thickness without a gap. For each participant, the scan lasted 300 s and 150 volumes were obtained.

### Data processing and analysis

Image preprocessing and statistical analyses were carried out using statistical parametric mapping software (SPM8, http://www.fil.ion.ucl.ac.uk/spm). The functional images underwent slice-timing correction and realignment for head motion correction. Data from the participants whose head motion exceeded 1.5 mm in the x, y, or z plane or whose head rotated more than 1.5° during scanning were excluded. After slice-timing correction and head-motion correction, the standard Montreal Neurological Institute (MNI) EPI template in SPM8 was used for spatial normalization with a resampling voxel size of 3×3×3 mm^3^. Then, the functional images were spatially smoothed using a Gaussian kernel with a full width at half maximum (FWHM) of 8 mm. Finally, linear trend subtraction and temporal filtering (0.01–0.08 Hz) were performed on the time series of each voxel to reduce the effects of low-frequency drifts and of physiological high frequency respiratory and cardiac noise by REST software (V1.6, http://resting-fmri.sourceforge.net) before subjecting the data to ALFF analysis [45].

### ALFF analysis

Analysis of ALFF was performed using REST software (http://resting-fmri.sourceforge.net). The filtered time series for each voxel were transformed to the frequency domain with a Fast Fourier Transform function, yielding a power spectrum. The square root of the power spectrum was calculated and then averaged across 0.01–0.08 Hz range at each voxel. This averaged square root was taken as the ALFF [31]. For standardization purposes, the ALFF of each voxel was divided by the global mean ALFF value to standardize data across participants, as done in many PET studies [46] and a standardized ALFF map of the whole brain was obtained.

### FC analysis

FC was examined with REST software (http://resting-fmri.sourceforge.net) by applying a seed-region approach [38]. In addition to standard preprocessing, resting-state data were corrected as described elsewhere [47]. Linear regression was used to correct for changes in ventricular, white matter, and global signals. In line with the prior hypothesis regarding the amygdala, we employed a seed-based approach whereby the mean time series for each participant was extracted from the bilateral amygdala formation as defined by automated anatomic labeling (AAL) implemented with wfu_PickAtlas software [48], [49]. The time series of raw functional magnetic resonance imaging (fMRI) data for each voxel were temporally band pass filtered (0.01–0.08Hz). Then, BOLD signal time courses were averaged for the left and right amygdala, separately, and correlated voxel-wise with the time course of each voxel in the entire brain. Finally, correlation maps were converted to z-values using Fisher's r-to-z transformation to enable group comparisons.

### Analyses of clinical variables in relation to ALFF and FC data

To investigate the relationship between ALFF, FC, and the severity of depressive symptoms in the patients, we computed Pearson's correlation coefficients between the ALFF, FC, and CES-D scores. In addition, some areas that showed altered FC to the amygdala were defined as ROIs, and the ALFF values in these ROIs were used to perform the correlation with the altered FC to amygdala in the MDD patients group.

### Statistical analysis

The primary analyses involved a comparison of patients and HCs in terms of (1) regional cerebral ALFF values, and (2) amygdala-seed resting FC by the seed voxel method. These analyses were performed across the whole brain using two-sample t-tests as implemented in SPM8 software, with age, gender, and educational level as nuisance covariates. In addition, within-group FC analysis was performed using one-sample t-tests as implemented in SPM8 software for each group. The results were interpreted with all statistical p map thresholds set to be cluster-corrected with family wise error *p*<0.05 (derived from an uncorrected *p*<0.001 and 50 extended voxels).

Three correlation analyses were performed in the MDD patients group using Pearson's correlation coefficient tests: the correlation of “altered ALFF values and the severity of depressive symptoms”, “altered FC to amygdala and the severity of depressive symptoms” and “altered FC to amygdala and ALFF values in regions where showed altered FC to amygdala”. In addition, demographic differences between the patient and control groups were analyzed using two- sample t-tests or X^2^ tests in all cases; *p*<0.05 was deemed statistically significant.

## Results

### Demographics

Demographic and clinical data of both HCs and MDD patients are summarized in Table 1. There were no significant differences between the MDD and HC groups in terms of gender, age, or years of education. MDD patients scored higher than HCs on the CES-D (t[65] = 13.35, *p*<0.001).

### Altered ALFF in patients with MDD

**Figure 1:**
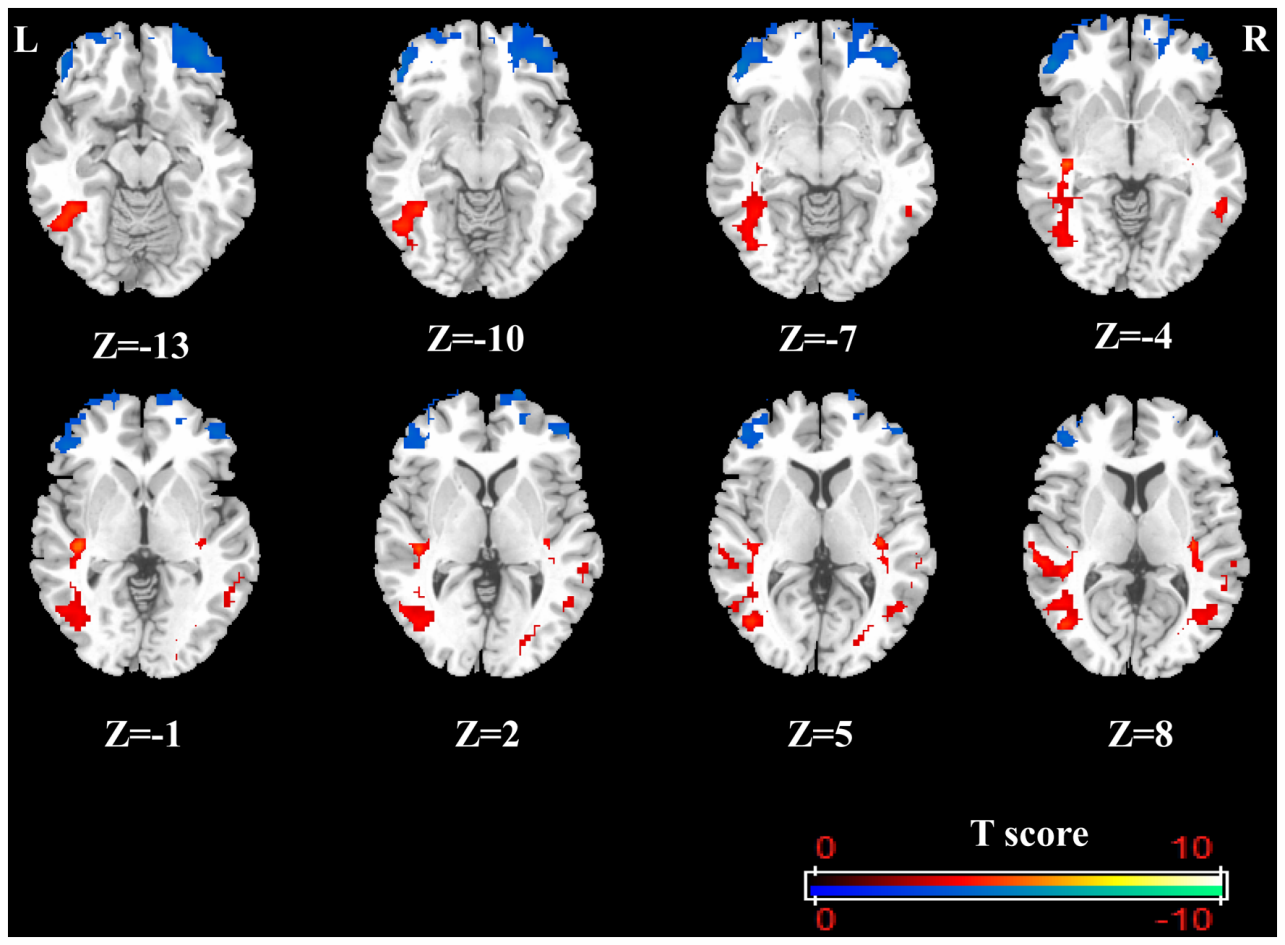
ALFF values using two-sample t-tests during resting-state. Regions showing decreased (blue) and increased (red) ALFF values in first-episode, drug-naive patients with MDD compared to HCs were at the threshold t >3.2, with correction for multiple comparisons applied at p<0.05 (cluster-corrected with family wise error). Color bar indicates the T score. (L =  left side; R =  right side)

In support of our hypothesis, patients with MDD showed significantly decreased ALFF bilaterally in the OFC compared to HCs (Figure 1 and Table 2). However, no ALFF changes were found in the amygdala, which was inconsistent with our hypothesis. In addition, significantly increased ALFF were found in the bilateral superior/middle/inferior temporal gyrus, extending to the bilateral insula, left fusiform gyrus, and left middle occipital gyrus (Figure 1 and Table 2).

**Table 2:** Regions that showed significant differences in ALFF values between the MDD and HC groups at rest.

Brani region	side	BA	Peak MNI coordinates (mm)			Cluster Size	Z score	*p* value*
			X	Y	Z			
*MDD<HC*								
OFC	L	11/47/10	−48	39	−6	195	4.37	<.001
OFC	R	11/47/10	36	45	−15	250	4.46	<.001
*MDD>HC*								
Insula	L	22/37/39	−39	−24	−3	592	5.34	<.001
Superior temporal gyrus								
Middle temporal gyrus								
Inferior temporal gyrus								
Middle occipital gyrus								
Fusiform gyrus								
Insula	R	13/39	36	−24	3	317	4.81	<.001
Superior temporal gyrus								
Middle Temporal gyrus								

Abbreviations: ALFF, amplitude of low-frequency fluctuations; BA, Brodman's area; L, left; R, right; MDD, patients with major depressive disorder; HCs, healthy controls; MNI, Montreal Neurological Institute;

Cluster-corrected with family-wise errors.

### Altered amygdala-OFC FC in patients with MDD

Both the HC and MDD groups showed positive amygdala connectivity to the basal ganglia, insula, parahippocampal/hippocampal gyrus, thalamus, anterior temporal cortex, subgenual cingulate cortex, and negative amygdala connectivity to the occipital and parietal cortices.

**Figure 2:**
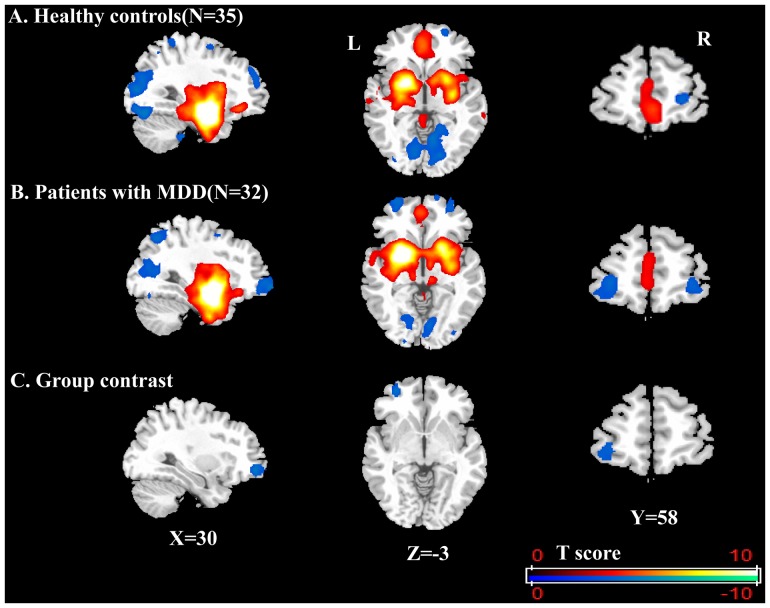
Left amygdala connectivity in HCs and MDD patients during a resting state. Using left amygdala as seed, negative FC (blue) and positive FC (red and yellow) were showed in HCs (A) and MDD patients (B). Compared with HCs, first-episode, drug-naive patients with MDD showed abnormal increased anti-correlation of activity between the left amygdala and left OFC (C). Color bar indicates the T score, and maps were at the threshold t >3.2, with correction for multiple comparisons applied at *p*<0.05 (cluster-corrected with family wise error). (L =  left side; R =  right side).

No differences were found between the HC and MDD groups for the right amygdala seeded FC analyses. However, for the left amygdala seed, negative FC between the amygdala and left OFC was apparent in MDD patients (Figure 2B) but not in HCs (Figure 2A). Relative to controls, the MDD participants showed abnormally enhanced anti-correlation of activity between the left amygdala and left OFC (Figure 2C, peak MNI coordinates X = −30, Y = 51, Z = −9, cluster-corrected *p*<0.005).

**Figure 3:**
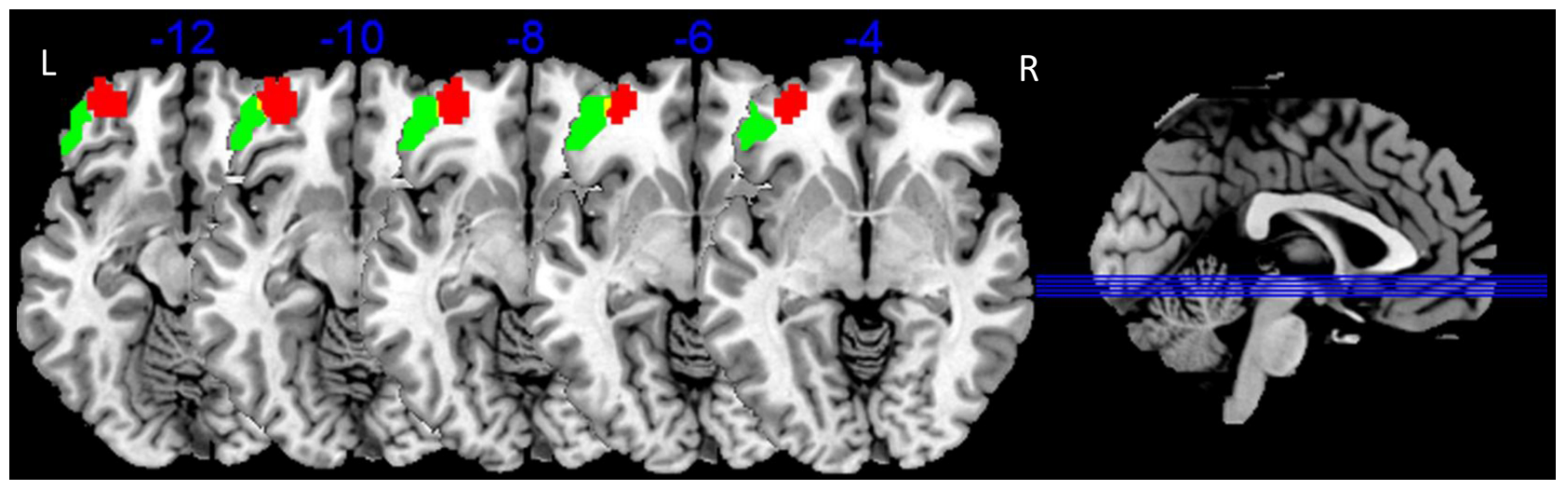
The overlap in the left OFC derived from ALFF results and FC results. (Green = altered ALFF in the left OFC; Red =  altered FC to amygdala in the left OFC; Yellow =  overlap; L =  left side; R =  right side).

The two brain regions in the left OFC, one showing decreased ALFF and one showing enhanced anti-correlation to the amygdala in MDD patients, did not spatially overlap. Only a few voxels were observed in both regions, as seen in Figure 3.

### Correlation analyses

There were no significant associations between CES-D scores and alterations in regional cerebral ALFF or FC values in patients with MDD. In addition, there was no significant correlation between the altered amygdala-OFC FC and ALFF values in the left OFC, where showing enhanced anti-correlation to the left amygdala in MDD patient group.

## Discussion

The current study investigated resting-state brain function by measuring ALFF and investigated amygdala-seeded FC in first-episode, medication-naive young adult patients with MDD. We found that, relative to HCs, patients with MDD showed significantly decreased ALFF values in the OFC bilaterally, and increased ALFF in the bilateral temporal lobe extending to the insular and left fusiform cortices. The decrease in ALFF values may be an indication of a functional deficiency. In addition, we found enhanced anti-correlation of activity between the left amygdala and left OFC in MDD patients, suggesting an indirect modulation of the affective network between the amygdala and the OFC. These findings indicate that compared to HCs, there is a concurrence of a hypoactive OFC and decreased OFC-amygdala FC in first-episode, medication-naive young adult patients with MDD at rest, supporting the notion that the OFC may play an important role in the pathophysiological processes of MDD.

### Changes in ALFF values

Prior resting-state neuroimaging studies have reported inconsistent results in relation to depression, including increased [13], [50], [51] or decreased [7]–[9] metabolism or blood flow in the OFC. The small sample size (<30) and variability in the cohorts' illness severity, diagnostic characteristics, and medication may have contributed to the inconsistency of these prior studies. To overcome these limitations, our study enrolled a relatively large cohort of patients who were all medication-naive and experiencing their first depressive episode. Our findings corroborate previous findings of decreased spontaneous activity in the OFC in patients with MDD [7]–[9]. Interestingly, human patients with OFC lesions show behaviors that in many ways resemble behaviors exhibited by patients with MDD, including depressed mood, anger, affective instability, irritability, and anxiety [52]. Previous structural and functional studies have consistently revealed abnormal OFC features in patients with MDD. For example, MRI morphometric studies have reported gray matter reductions in the OFC of patients with MDD [10]–[12]. Postmortem studies further provided morphological evidence for the involvement of cell atrophy in the OFC in MDD [53], [54]. Reduced metabolism of the OFC in depressed patients has also been reported in several studies using different brain imaging techniques including magnetic resonance spectroscopy [55], PET [7], [8] and single-photon emission computed tomography [9]. In addition, severity of depressive symptoms correlates inversely with OFC activity in MDD patients [8], [13]. Given that anhedonia is a classic symptom of depression, it is interesting to note that the OFC has been implicated in reward valuation [56] and hedonic experience [57]. These imaging findings provide converging evidence supporting the view that MDD involves OFC dysfunction.

Neurotransmitter studies also support the important role of the OFC in MDD. For example, reduced central serotonin and catecholamine neurotransmission (produced by tryptophan depletion and alpha-methyl-para-tyrosine administration, respectively) have been shown to decrease metabolism in the OFC and trigger depressive relapse in remitted patients with MDD [58]–[60]. Both serotonergic and catecholaminergic transmission appear necessary for optimal PFC function, and depletion of these neurotransmitters impairs OFC functioning [61], [62]. These findings further suggest that OFC dysfunction may underlie vulnerability to depression, perhaps due to impaired emotional and cognitive regulation. Whether the reduced synchronicity in the OFC of our patient group was the result of depleted central serotonin and/or catecholamine neurotransmission is unknown and remains an important question for future study.

Our findings of increased spontaneous activity in the bilateral temporal lobe of patients with MDD are consistent with recent rs-fMRI studies examining depression [63], [64]. A recent study also found abnormal ALFF in the fusiform and temporal regions in MDD patients [36], which may reflect perturbations in neural networks related to social functioning and emotional processing. The fusiform gyrus, part of the visual recognition network, is thought to be involved in the perception of emotions during the presentation of facial stimuli [65], [66]. Altered responsiveness to facial emotional stimuli has been suggested as a possible biomarker for the early diagnosis of MDD [67].

However, we did not observe any ALFF changes in the amygdala in patients with MDD compared with healthy controls. This null finding is inconsistent with prior neuroimaging evidence of apparently increased amygdala activity in MDD patients [14]–[16]. The reason for the inconsistency could arise from differences in experimental design. Those studies are based on event-related fMRI, whereas our study uses resting-state fMRI.

### Amygdala-OFC FC

In the current study, we demonstrated that, relative to the HC group, patients with MDD showed significantly enhanced anti-correlation of activity between the left amygdala and left OFC, suggesting increased top-down inhibition of the OFC on the amygdala. Previous imaging studies have suggested an inhibitory effect of the OFC on the amygdala [17]–[19]. Urry and colleagues found evidence of inverse coupling of the amygdala and ventromedial PFC, the portion of the PFC that includes the OFC (i.e. BA10), during regulation of negative emotion [21]. Thus impaired functioning of this region (OFC/ventromedial PFC) may disrupt top-down inhibition of the OFC on the amygdala, which may then compromise one's ability to regulate cognition and emotion, especially negative emotion. Indeed, Versace et al. suggested recently that elevated left amygdala-OFC FC in subjects viewing sad stimuli could be used as a depression state marker [24]. Using dynamic causal modeling, a technique for examining inter-regional influences [68], Almeida et al. found that, relative to controls, patients with MDD had significantly greater negative left-sided top-down connectivity between the OFC and amygdala [25]. Furthermore, Tang et al. observed decreased FC between the amygdala and the left ventral PFC in treatment-naive patients with MDD [38]. The present study corroborates and extends these earlier findings by demonstrating that patients with MDD, when at rest, exhibit abnormal left amygdala-OFC FC, suggesting an indirect modulation of the affective network between the amygdala and the OFC, and possibly increased top-down inhibition of the OFC on the amygdala. Our FC results extend our understanding of the integration of the amygdala and OFC in MDD patients in a resting state.

Together with these prior studies, the present results from both ALFF and FC analysis suggest that impaired OFC functioning may be an important factor that could affect the top-down inhibition of the OFC on the amygdala. Additionally, the enhanced anti-correlation of activity between left-side OFC and amygdala possibly reflects dysfunction of the amygdala-OFC network and may represent a pathological process of MDD. The enhanced anti-correlation between left-side OFC and amygdala seen in MDD patients but not in HC could represent a maladaptive process of MDD. In our results, the two regions that show decreased ALFF in the left OFC, but abnormal FC to left amygdala in the left OFC do not substantially overlap, which could motivate studies of the correlation between these two features in the future.

### Limitations

There are several limitations of this study. First, as patients with MDD were already experiencing acute episodes, future studies of individuals at risk for MDD may be warranted to elucidate the changed resting brain function in the affective network associated with a predisposition to MDD. Secondly, we cannot ensure the clinical consistency of MDD patients because the patients are younger and in an earlier phase of their illness, and 12.5%–30% of MDD patients may develop bipolar disorder in subsequent years [69]. In addition, although the ALFF has been increasingly used to measure spontaneous neural activity [33], its exact neurophysiological basis remains unclear. The underlying reasons for reduced OFC spontaneous activity in patients with MDD need to be discerned in future studies.

## Conclusions

The current study reveals significantly reduced resting-state ALFF but abnormally enhanced anti-correlation of activity between the left-side OFC and the amygdala in first-episode, medication-naive young MDD patients compared with healthy controls. Decreased ALFF in the OFC may reflect the hypo-functioning of negative emotion regulation in the affective network, which could be a hallmark of major depression. Enhanced anti-correlation of activity between the OFC and amygdala may reflect dysfunction of the amygdala-OFC network and additionally represent a pathological process of MDD.
